# Understanding dynamics of coherent Ising machines through simulation of large-scale 2D Ising models

**DOI:** 10.1038/s41467-018-07328-1

**Published:** 2018-11-27

**Authors:** Fabian Böhm, Takahiro Inagaki, Kensuke Inaba, Toshimori Honjo, Koji Enbutsu, Takeshi Umeki, Ryoichi Kasahara, Hiroki Takesue

**Affiliations:** 1 NTT Basic Research Laboratories, NTT Corporation, 3-1 Morinosato Wakamiya, Atsugi, Kanagawa 243-0198 Japan; 2 Institut für Optik und Atomare Physik, Technische Universität Berlin, Hardenbergstrasse 36, 10623 Berlin, Germany; 3 NTT Device Technology Laboratories, NTT Corporation, 3-1 Morinosato Wakamiya, Atsugi, Kanagawa 243-0198 Japan

## Abstract

Many problems in mathematics, statistical mechanics, and computer science are computationally hard but can often be mapped onto a ground-state-search problem of the Ising model and approximately solved by artificial spin-networks of coupled degenerate optical parametric oscillators (DOPOs) in coherent Ising machines. To better understand their working principle and optimize their performance, we analyze the dynamics during the ground state search of 2D Ising models with up to 1936 mutually coupled DOPOs. For regular as well as frustrated and disordered 2D lattices, the machine finds the correct solution within just a few milliseconds. We determine that calculation performance is limited by freeze-out effects and can be improved by controlling the DOPO dynamics, which allows to optimize performance of coherent Ising machines in various tasks. Comparisons with Monte Carlo simulations reveal that coherent Ising machines behave like low temperature spin systems, thus making them suitable for optimization tasks.

## Abstract (web summary)

Coherent Ising machines constitute a possible way to solve Ising models, but there are still open questions about their dynamics. In this work, the authors simulate regular and frustrated 2D lattices, highlighting the role of optical pump power and freeze-out effects for solving optimization problems.

## Introduction

Many problems in mathematics, statistical mechanics or computer science are known to be NP (non-deterministic polynomial-time)-hard or even NP-complete, which makes it difficult to solve them on conventional computer systems. Even with advances in algorithms and the processing power of modern supercomputers, such computationally hard problems can often not be solved exactly in a reasonable time, which presents a major challenge for many real-world applications such as finance, drug discovery, cryptography, machine learning and optimization and analysis of large networks^1–3^. Interestingly, many of these problems can also be mapped onto the Ising model and solved by finding the ground state of the Ising Hamiltonian^4^. This allows to find approximate solutions with various heuristic methods, such as quantum annealing or adiabatic quantum computing. In these analog computing schemes, the Ising model is implemented in physical systems instead of conventional digital computers. The binary spin states of the Ising model are then represented by a variety of physical systems, including superconducting circuits^5–7^, trapped ions^8^ or CMOS (complementary metal–oxide–semiconductor) devices^9^. The energy of these artificial spin networks is proportional to the Ising Hamiltonian so that they will naturally evolve to the Ising ground state. Compared to conventional Markov chain Monte Carlo (MCMC) methods, where performance often suffers from long transients or trapping in local energy minima^10^, these novel computing schemes promise a significant speed-up of calculation time.

A particular concept that has attracted recent interest is to build optical artificial spin networks using degenerate optical parametric oscillators (DOPOs)^11^. In these coherent Ising machines (CIMs), spins are represented by the optical phase of the DOPOs, which is either 0 or *π* relative to the pump laser driving the DOPO generation process. Implementation of the Ising Hamiltonian is then achieved by mutual optical coupling either with delay interferometers^12,13^ or opto-electronic measurement feedback schemes^14,15^. Contrary to typical quantum annealing devices, opto-electronic CIMs can implement and simulate arbitrary coupling topologies for large spin systems, and they have been successfully used to study different large-scale problems from one-dimensional (1D) spin chains^12,13^ to NP-hard optimization problems^14,15^. By taking advantage of fast field-programmable gate array (FPGA) hardware, CIMs have also demonstrated a significant speed-up for dense graph structures over conventional methods such as simulated annealing^14^. However, while some efforts have been made to understand the operating principle of CIMs^16–20^, there is still considerable ambiguity regarding how they evolve to their lowest energy state and what factors can limit the calculation performance. Among the open questions are the apparent scaling of the calculation performance with the optical pump power or the effective temperature of the implemented spin system^13^.

In this work, we present a detailed analysis of the CIM dynamics and performance in finding the ground state for large-scale two-dimensional (2D) Ising models. We implement and simulate different regular and frustrated 2D lattices with a set of up to 1936 artificial spins and observe the structure of the spin domains and its dynamics with different operating parameters. We demonstrate that a CIM can find the ground state even for large frustrated or disordered graph structures on fast timescales of only a few milliseconds. We also identify a common effect, in which the DOPO dynamics freeze-out and prevent the machine from reaching the ground state as a dominant performance inhibiting factor. By analyzing the dynamics of the DOPO network, we find how this effect is linked to the calculation success and the machine’s operating parameters. Based on this finding, we present ways to optimize CIMs, which can be used to improve the performance in various tasks. By comparing our results against Monte Carlo simulations, we also extract a temperature estimate and demonstrate that a DOPO network behaves like a low-temperature spin system, even though it operates at room temperature, thus showing the inherent suitability of CIMs for optimization tasks.

## Results

### DOPO dynamics during ground state search

**Fig. 1:**
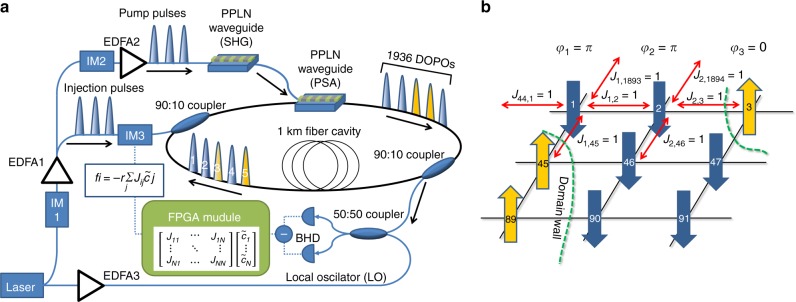
Experimental setup and exemplary coupling scheme for 2D Ising models. **a** Schematic of the experimental setup for a coherent Ising machine with an opto-electronic measurement feedback system using 1936 DOPOs. IM intensity modulator, EDFA erbium-doped fiber amplifier, PPLN waveguide poled lithium niobate waveguide, BHD balanced homodyne detection, SHG second harmonic generation, PSA phase-sensitive amplifier. **b** Coupling topology *J*_*ij*_ for the ferromagnetic 2D Ising model with 1936 spins implemented in the coherent Ising machine. The spins are indexed using a typewriter ordering with periodic boundary conditions and coupled to their nearest neighbors. Spins are distinguished by the optical phase *φ* of each DOPO into spin up (yellow, *φ* = 0) or spin down (blue, *φ* = *π*)

A CIM generates an artificial spin network from DOPOs circulating in a ring fiber cavity. These optical states have a binary phase that is either *φ*_*j*_ = 0 or *φ*_*j*_ = *π* relative to the pump laser driving the DOPO generation process. It has been shown that the energy of such a DOPO network is proportional to the Ising Hamiltonian

1 $$H_{{\mathrm{Ising}}}(\sigma ) = - \frac{1}{2}\mathop {\sum}\limits_{i < j} {\kern 1pt} J_{ij}\sigma _i\sigma _j,$$

where the optical phase *φ*_*j*_ = {0, *π*} represents the spin state *σ*_*j*_ = {−1, 1}^17^. To implement the coupling term *J*_*ij*_, the individual DOPOs are optically coupled through an opto-electronic feedback scheme in which a FPGA generates the feedback signal and a push–pull modulator modulates the optical phase and amplitude of a train of external injection pulses (see Fig. 1a; further details are given in the Methods section). This enables CIMs to find the ground state of arbitrary Ising problems by the minimum gain principle^16,17^. When the pump power for the DOPO generation process is turned on, the gain in the ring cavity exceeds the threshold and the DOPO intensity starts to increase. The configuration that minimizes the Ising Hamiltonian simultaneously minimizes the gain above the threshold and the DOPOs will end in the correct state after reaching the saturation amplitude. To perform a single calculation step the pump pulse power is thus first turned on to a constant value and then turned off after the final configuration is reached. To understand the DOPO dynamics during this process, we implement three different 2D graph structures with nearest neighbor coupling.

We investigate a 44 by 44 square lattice structure with anti-ferromagnetic coupling (*J*_*ij*_ = −1), as well as a triangular lattice and a random lattice. Among the three graphs, the square lattice is the only Ising problem with an exact analytical solution^21^. For ferromagnetic coupling *J*_*ij*_ = 1, the ground state is reached when all spins are aligned in one direction. For anti-ferromagnetic coupling, the ground state is a checkerboard pattern with spins alternating between the spin-up and spin-down configurations. Due to this trivial nature, the 2D square lattice serves as a common benchmark for software solvers and is also one of the simplest systems with a phase transition, making it a paradigmatic model for many systems in statistical mechanics.

**Fig. 2:**
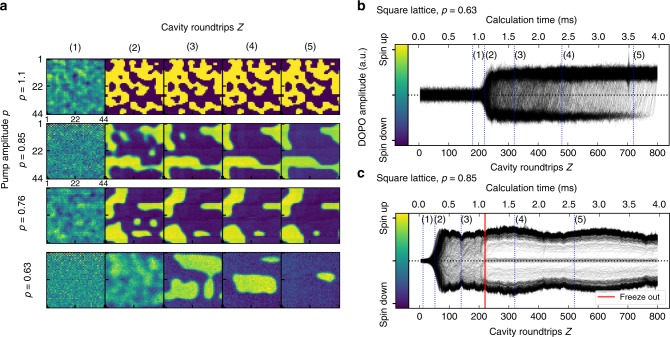
Time evolution of the DOPOs during ground state search for the regular 2D lattice. **a** Snapshots of the time evolution of each DOPO’s in-phase quadrature amplitude *c*_*i*_ at different pump amplitudes *p* = {1.1, 0.85, 0.76, 0.63} visualized in the regular lattice structure. Blue indicated the spin-down and yellow the spin-up configuration (colorbar in (**b**)). The DOPO amplitude grows from a squeezed vacuum state (1) to the saturation amplitude (2). As the amplitude grows, domains with distinct domain walls are formed. As the calculation continues, the domain walls start to move and domains start to shrink until eventually all domains have evaporated and the machine has reached the ground state. **b**, **c** Exemplary time evolution for the in-phase quadrature amplitude of each DOPO at *p* = 0.63 (**b**) and *p* = 0.85 (**c**). In (**b**), the ground state is eventually reached after around 800 cavity roundtrips. The red line in (**c**) indicates the point where evolution to the ground state prematurely stops and the domains remain frozen in their final configuration (i.e., freeze-out)

For the square lattice, we show the time evolution during the calculation cycles for each DOPO in Fig. 2. The figure shows both the time traces (Fig. 2b) and (Fig. 2c) and snapshots (Fig. 2a) of the resulting 2D domain structure for each of the 1936 DOPOs. For the snapshots, the in-phase quadrature component $\tilde c_i$ is color coded so that blue indicates cases where the phase relative to the pump laser is *φ*_*j*_ = *π* (or spin down *σ* = −1) whereas yellow indicates a phase of *φ*_*j*_ = 0 (or spin up *σ* = 1). Note here that the DOPO amplitudes are multiplied with a checkerboard pattern, which transforms the problem into the ferromagnetic case. While this does not change the result of the measurement, it increases visibility and helps to better understand the domain dynamics. For all measurements, we varied the normalized pump amplitude of the pump pulses using values of *p* = 1.1, *p* = 0.85, *p* = 0.76 and *p* = 0.63 (equivalent to respective optical powers of 25.1, 23.8, 23.4 and 22.6 dBm) by changing the gain of the intensity modulator (IM2). The injection power was left constant at −34.6 dBm. The normalized pump amplitude *p* is given in units relative to the threshold of the uncoupled DOPOs without injection pulses. With optical coupling, the threshold of the pump amplitude is shifted below that for the uncoupled DOPOs by the additional optical gain from the injection pulses, so that *p* = 1 is not the threshold value for the coupled DOPOs.

For all measurements, calculation starts from a random spin state (snapshot (1)), since the DOPOs are in a squeezed vacuum state below the threshold^17^. As the calculation continues, the ground state search is performed in a two-stage process, namely a growth and a saturation stage^16^. In the growth stage, the DOPO amplitude increases over time and the artificial spins start to self-organize into a domain structure with clearly defined domain walls (snapshot (2)). In the saturation stage, the DOPOs have reached their saturation amplitude due to pump depletion. During this stage, domain walls will start to radially move inward so that domains shrink and evaporate over time (snapshots (3)–(5) for *p* = 0.63). We observe that domain walls typically move at a speed corresponding to their curvature, so that strongly curved walls move faster. Therefore, small domains will vanish first, which was predicted for CIMs with optical coupling^16^. It has been shown that the domain wall speed scales with *v* ∝ 1/*r* over the domain radius, which can also be observed in our experiments.

For the lowest pump amplitude at *p* = 0.63, this process continues until the ground state has been reached. We can thus verify that through the domain evaporation process, CIMs are able to successfully perform the ground state search for square lattice problems for a large number of artificial spins. Furthermore, the fast DOPO dynamics allow for quick computation of the ground state energy. From the time series in Fig. 2b, we observe that the DOPOs have reordered themselves in the ground state at *Z* = 800 cavity roundtrips. The equivalent calculation time is at around 4 milliseconds, which is similar to timescales that have been reported for CIMs in MAXCUT optimization problems^14,15^. In this short timeframe, the FPGA performs around 1.5 million individual spin updates at a rate of one update per 2.5 nanoseconds. Compared with software-based simulations, we find that spin updates from the FPGA occur on timescales similar to those of highly optimized parallel solvers running on graphics processing units (GPUs)^22,23^. While software simulations benefit from update parallelization due to the simple nearest neighbor coupling in the 2D Ising model though, CIMs show superior performance for Ising problems with dense graph structures^14^.

### Impact of dynamical freeze-out on calculation performance

Theoretical predictions suggest that, given enough calculation time, the ground state can always be reached for regular lattices with coherent Ising machines^16^. However, we find that in many cases, the domain evolution will freeze at a certain point (red line in Fig. 2c). Beyond this point in time, the amplitude of each DOPO will remain unchanged for the rest of the calculation and the domain structure will remain frozen in its final configuration (snapshots (4)–(5) for *p* = 0.85). To better understand the origin of this freeze-out process and develop strategies for optimizing performance, we further investigate the DOPO dynamics and the influence of the operating parameters.

**Fig. 3:**
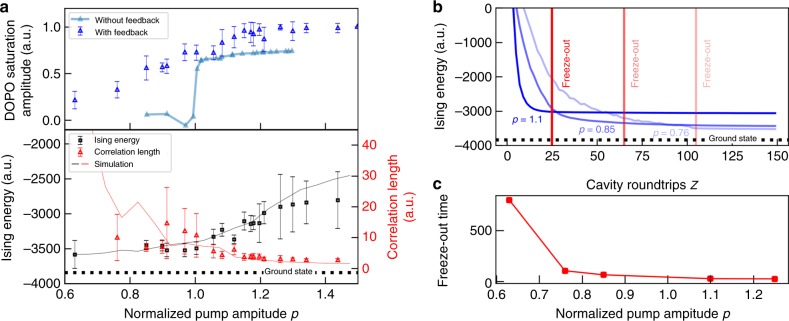
Effect of dynamical freeze-out on calculation performance in the regular 2D lattice. **a** Saturation amplitude (blue and teal triangles), Ising energy (black squares) and correlation length (red triangles) for the regular 2D lattice after 1000 cavity roundtrips. The solid lines are obtained from numerical simulations. Error bars represent the standard deviation of 50 trials. **b** Time evolution of the Ising energy during the ground state search for pump amplitudes *p* = {1.1, 0.85, 0.76}. Estimated freeze-out times are represented by red lines. **c** Scaling of the freeze-out time with pump amplitude *p*

From the time evolution data, we can observe that the pump amplitude has a crucial influence on the domain dynamics. During the growth stage, higher pump amplitudes evoke domain structures consisting of many small domains, resulting in a high number of domain walls. For lower pump amplitudes closer to the threshold, the time to reach saturation is longer and the size of the domains at the end of the stage is in turn increased, bringing the system closer to the ground state. Likewise, during the saturation stage, the speed of domain wall movement is typically higher for large pump amplitudes, which agrees well with theoretical predictions^16^. This is also reflected in the time evolution of the Ising energy (see Fig. 3b). For large *p*, the energy will quickly decrease until the point of freeze-out is reached, while for low *p*, the energy decreases more gradually and ultimately reaches closer to the ground state before freeze-out occurs. This slowdown is linked to a general slowdown in the DOPO dynamics, where the time taken to reach saturation amplitude or to flip the DOPO amplitude changes with the pump amplitude. This is due to the link between the dynamical timescales and the DOPO saturation amplitude^14,16^, which can be controlled by changing the strength of the pump or injection pulses in our setup. However, since we leave the injection power fixed in the experiments, the saturation amplitude is only controlled by the pump amplitude, leading to a direct link between ground state search performance and the pump amplitude.

We also find that the time until freeze-out increases with decreasing pump amplitude. We estimate the freeze-out time from the time series (indicated by the red lines in Fig. 3b) and find that there is a significant increase towards the threshold (see Fig. 3c). This delays the freeze-out long enough for a significant number of computations to reach the ground state before freeze-out occurs. This is probably caused by the higher ratio of the feedback to the DOPO amplitude towards the threshold. Specifically, a higher injection power will increase the probability of the spin state changing in the saturation stage, since a minimal injection amplitude is required to change the sign of the in-phase quadrature amplitude of a single DOPO. We checked the behavior at lower injection strengths by inserting an additional attenuator in the injection line and observed a corresponding decrease in the freeze-out delay. This results in a distinct scaling of the final Ising energy with the pump amplitude. Figure 3a shows the Ising energy of the final state in relation to the ground state in a pump range from *p* = 1.45 to *p* = 0.63 after 1000 roundtrips. Far away from the DOPO threshold, the final state will reach an average of 75% of the ground state energy before freeze-out, while closer to the threshold, the average energy will gradually decrease to around 95% at *p* = 0.63. A similar scaling of the Ising energy with the pump amplitude was also reported for 1D spin chains^13^. Through our analysis, we can now identify dynamical freeze-out as the primary cause for this behavior. We also derive the correlation length *l*_0_ of the spin set by fitting the 2D autocorrelation function with an exponential function exp(*x*/*l*_0_). For high pump amplitudes, the large number of small domains will lead to the fast decay of the autocorrelation function and hence a small correlation length. As the pump amplitude decreases, the larger and more evenly shaped domains result in an increase in the correlation length. We compare this pump amplitude scaling of the energy and correlation length with numerical simulations of a time discrete stochastic differential equation model^17^ and find that they are in overall good agreement (solid lines in Fig. 3a), corroborating the negative effect of freeze-outs on the calculation performance at higher pump amplitudes. We can thus conclude that both the slowdown in dynamics and the higher ratio of injection to DOPO amplitude are important contributing factors to decrease the frequency of freeze-outs and hence improving the performance of the CIM.

### Temperature estimation and Monte Carlo simulations

**Fig. 4:**
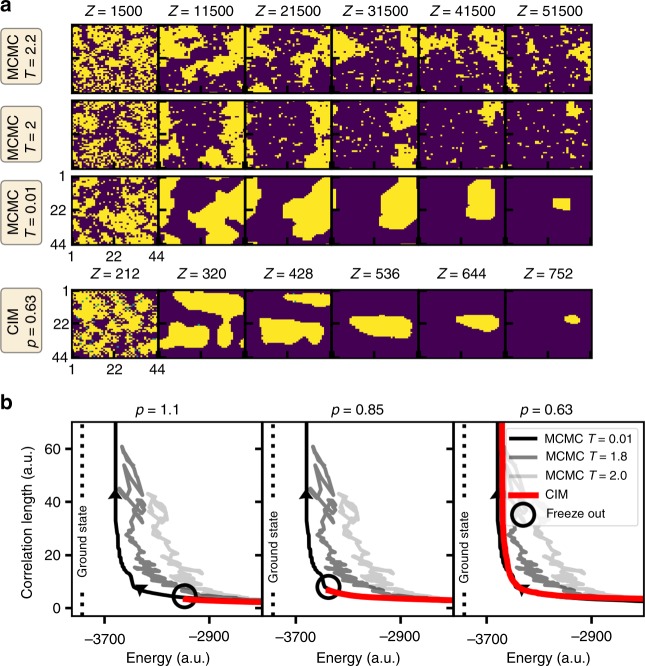
Comparison of Monte Carlo simulation (MCMC) and coherent Ising machine (CIM) during the ground state search. **a** Snapshots of the spin configuration (blue: spin down, yellow: spin up) as it approaches the ground state obtained by Monte Carlo simulations (MCMC) at different temperatures *T* = {0.01, 2.0, 2.2} and by the CIM at *p* = 0.63. **b** Time evolution of energy and correlation length during the ground state search for both Monte Carlo simulations at different temperatures (black and gray lines) and the coherent Ising machine (red line) at pump amplitudes *p* = {1.1, 0.85, 0.63}. The arrows show the direction of the trajectories as the ground state is approached. Freeze-out points are indicated by black circles

As to the actual cause of the freeze-out process, we consider two effects that are typically observed in Monte Carlo methods, i.e., trapping in local energy minima and high spin temperatures. Local energy minima for instance are spin configurations, which are hard to escape from once they are reached. In the 2D square lattice, such local energy minima are typically associated with parallel domain walls. From the snapshots however, we find that this is typically not the case for frozen states (see Fig. 2a), thus ruling out local energy minima as a cause for the freeze-out process. High temperatures of the spin system on the other hand cause the spins to reach an equilibrium state which is far away from the actual ground state. In quantum annealing hardware, it has been shown that the non-equilibrium dynamics in the annealing process can typically be associated with an effective temperature^24^, hence requiring long annealing times to reach a low effective temperature. To relate the freeze-out process in the coherent Ising machine to an effective temperature, we compare the ground state search to Markov chain Monte Carlo simulations using the Metropolis–Hastings algorithm at different temperatures in Fig. 4^25^. Starting from a random spin state, we find that Monte Carlo simulations at low temperatures exhibit the same domain clustering process as the CIM (see Fig. 4a): smaller domains will evaporate or merge to form larger ones, until the ground state is reached. At higher temperatures in the Monte Carlo simulation, temperature-induced spin flips will lead to more irregularly shaped domains and create small temporary domains. As the phase transition of the square lattice model is approached, these temperature-induced domains will grow in size and the correlation length will increase.

For a quantitative comparison of the domain clustering dynamics, we consider the time evolution of the energy and the correlation length. Since it can be difficult to relate steps in the Monte Carlo simulation to those in the coherent Ising machine, we compare the trajectories in the phase space of the energy and the correlation length (see Fig. 4b). These trajectories visualize the relationship between energy and correlation length as the simulation approaches the ground state. We find that different temperatures in the Ising model result in different trajectories in the Monte Carlo simulations (black and gray lines in Fig. 4b). At low temperatures (black line) as the energy decreases, the correlation length will remain almost constant at the beginning and then rapidly increase and diverge as the energy approaches the ground state. For higher temperatures close to the phase transition in the 2D Ising model, the correlation length is higher, leading to a deviation from the low-temperature trajectory as the final state is approached (gray and light gray lines). For the CIM, we find that the trajectories (red curves) agree very well with the low-temperature behavior of the Metropolis algorithm. This corroborates the similarities seen in the 2D visualization in Fig. 4a. Interestingly, this is always the case for different pump amplitudes. Even when freeze-out occurs, there is no deviation from the low-temperature trajectory. This indicates that the freeze-out can be interpreted as a frozen transient state along the trajectory rather than a temperature-induced effect, as was previously assumed^13^. In fact, our analysis reveals that the CIM best mimics the behavior of the Ising model at very low temperatures, although the machine is operating at room temperature. This makes the machine inherently suitable for simulating low-temperature spin systems, which is an important prerequisite for solving optimization problems.

### CIM performance for frustrated 2D graphs

From the analysis of the square lattice model, it becomes apparent that the dynamics of a CIM play a crucial role in finding the solution to a problem. In accordance with previous theoretical predictions, a clustering of the spins into domains could be observed in the time series analysis that allows the machine to progress to the ground state. However, the appearance of freeze-out process is an effect that goes beyond the theoretical models and explains the link between the machine performance and the pump amplitude. The relation between the dynamics of individual DOPOs and the timescales of the freeze-out presents a way to optimize machine performance and avoid freeze-outs. If the saturation amplitude is low and the injection to pump power ratio is high, we showed that the ground state can be found, even for large spin numbers. The comparison to Monte Carlo simulations further shows how freeze-out effects are caused neither by local energy minima nor temperature, thus showing the inherent suitability of CIMs for optimization problems.

**Fig. 5:**
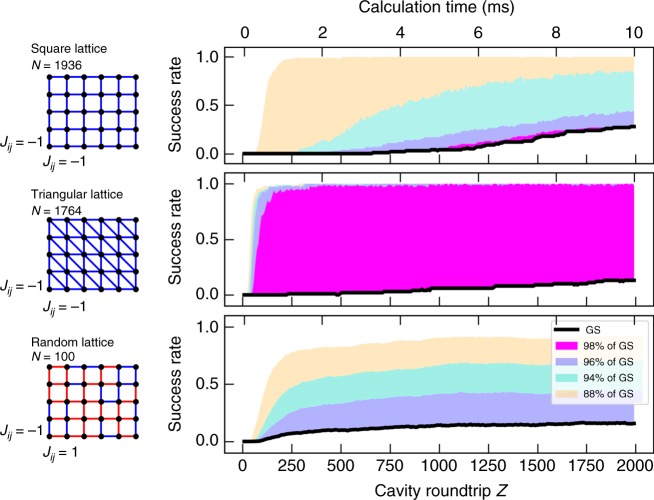
Success rate to reach the ground state for various 2D graphs. Graph structure and time evolution of the success rate to reach the ground state for the regular lattice, triangular lattice and 2D random lattice. For the graph structures, red links indicate ferromagnetic coupling and blue links indicate anti-ferromagnetic coupling. The different colors for the success rate indicate the frequency of cases where 98% (magenta), 96% (blue), 94% (turquoise) and 88% (orange) of the ground state energy was reached

**Fig. 6:**
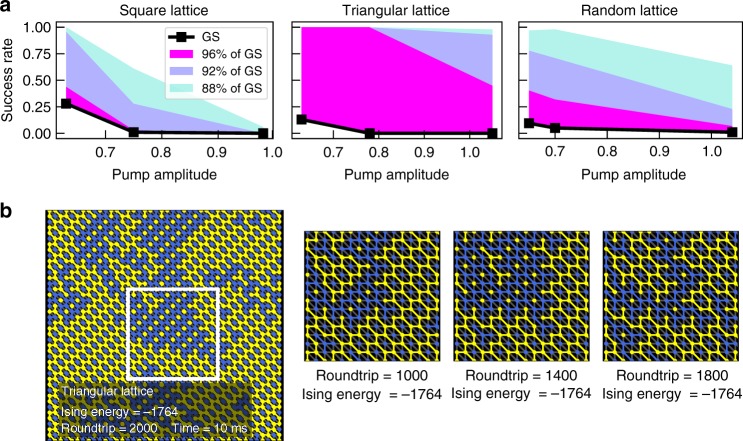
Pump amplitude scaling of success rate and degenerate ground states. **a** Scaling of the success rate with the pump amplitude for all three graph structures. The different colors for the success rate indicate the frequency of cases where 96% (magenta), 92% (blue) and 88% (turquoise) of the ground state energy was reached. **b** Snapshot and close ups of a successful ground state search in the triangular lattice. Blue and yellow circles show when spins are in the up and down configuration (blue: down, yellow: up). Blue and yellow links indicate frustrations in the lattice due to competing spin interactions, whereas gray links show cases where neighboring spins have different signs. As the calculation progresses, a jumping between different degenerate ground states can be observed

To show how freeze-out processes affect calculation performance in general, we have evaluated the overall success rate of the CIM in solving different problems. Figure 5 shows the time evolution of the success rate for the square lattice problem (*p* = 0.63), as well as for the triangular lattice (*p* = 0.63) and for 2D random lattices (*p* = 0.65). For each graph, 100 trials were performed to estimate the success rate. For the square lattice, a success rate of 28% is achieved after 2000 roundtrips, while the remaining states are either still approaching the ground state or stuck in freeze-out. The effect of freeze-outs becomes apparent when we compare the success probability for different pump amplitudes (see Fig. 6a). As the pump amplitude increases, the probability to reach the ground state quickly drops to zero as more and more states experience freeze-out.

As a more challenging benchmark, we also consider the triangular lattice and the 2D random lattice. Both are instances, where competing spin interactions lead to lattice frustrations. These result in complex patterns in the ground state and present a challenge for Monte Carlo simulations to obtain the ground state for large lattices. Since simple algorithms like the Metropolis–Hasting algorithm are unlikely to find the ground state due to critical slowdown or local energy minima, more sophisticated solvers such as cluster algorithms or simulated annealing are typically required. In the triangular lattice, every node has two additional anti-ferromagnetic links to its diagonal neighbors, which cause the lattice frustration. We implement the triangular lattice in a 42 by 42 lattice with a total of 1764 spins. The CIM reaches a success rate of 13% after 2000 cavity roundtrips. In the remaining calculation, all fall within 96% of the correct solution, which presents a good approximation to the ground state. For a successful calculation, we can clearly observe how the frustrations form in the lattice. Figure 6b shows several snapshots of a single instance, where regular frustrations in the form of hexagonal patterns develop in the lattice as the machine reaches the ground state (frustrations are indicated by yellow and blue lines). It is interesting to note that the frustrations will continually move through the lattice, so that the machine switches between different degenerate ground states. In the 2D random lattice, similar frustrations are caused by randomly selecting between anti-ferromagnetic and ferromagnetic coupling for each link. For the 2D random lattice, we randomly generate 20 different 10 by 10 lattices. A success rate of 16% was achieved in the median over 20 lattices. The ability to reach the ground state in a relevant number of calculations demonstrates that CIMs can effectively escape local energy minima. This enables them to find the correct ground state within only a few milliseconds, even when lattice frustrations are present. It is also important to note here that the pump amplitude was kept constant throughout the experiment for the sake of simplicity. As demonstrated in previous studies, we would expect a higher success rate if the pump amplitude was gradually increased^14^. Like in the regular lattice, we also observe the expected scaling of the success rate with the amplitude due to the freeze-out process for the triangular and the 2D random lattice (see Fig. 6a). This demonstrates the general nature of the freeze-out process to affect the performance of coherent Ising machines and how it can be controlled by the DOPO dynamics.

## Discussion

In conclusion, we have analyzed how coherent Ising machines evolve to their lowest energy state during a ground state search of large-scale 2D Ising models with up to 1936 artificial spins. Through time series analysis, we were able to verify previous predictions of DOPO domain dynamics and could show that the machine is able to find the ground state in a relevant number of calculations within timescales of only a few milliseconds, even in the presence of lattice frustrations. Contrary to previous studies, we were able to identify a general performance inhibiting mechanism, in which the DOPO dynamics will freeze-out and stop further evolution to the ground state. We could show how this effect is linked to the machine’s parameters, which explains the scaling of calculation performance with the optical pump power that was reported in previous studies. We demonstrate how the performance can be optimized by decreasing the DOPO amplitude close to the cavity threshold and expect that our findings can be used to improve the performance of the machine in various applications.

Further analysis of the freeze-out effects reveals that this effect is induced neither by trapping in local energy minima nor by a high spin temperature. A comparison with Monte Carlo simulations shows that, regardless of the operating parameters, the CIM appears to solve the Ising model at low temperatures. From our analysis, we conclude that this is a general property of the machine that applies to any kind of Ising problem. This is fundamental in understanding solutions to more complex problems and also makes the machine suitable for optimization problems such as MAXCUT and the traveling salesman or graph coloring problem. The low-temperature behavior is also different from quantum annealing, where freeze-outs are associated with an effective spin temperature. While this corroborates the robustness of the calculation results in CIMs against noise and power fluctuations, it also opens new questions about the behavior of coherent Ising machines at finite temperatures. Although small amounts of noise appear to have little effect on the final calculation result, strong optical noise can be expected to induce randomized flips in the phase of the DOPOs, similar to temperature-induced spin flips in the Ising model. As for Monte Carlo simulations or simulated annealing, temperature could be used to escape local energy minima. Likewise, the constantly induced change in the DOPO state could be used to further reduce the frequency of freeze-outs and lead to an overall increase in the performance of CIMs. Together with the inherent speed of the optical system, this makes the coherent Ising machine a versatile and fast alternative to software-based simulations on digital computers.

## Methods

### Coherent Ising machine

The coherent Ising machine implements the Ising Hamiltonian by employing time-multiplexed DOPOs that circulate inside a 1 km long fiber ring cavity (see Fig. 1a). The DOPOs are generated from a pulse train consisting of 40 ps-long pump pulses with 1 ns pulse interval times, modulated from a CW laser by an intensity modulator (IM1), with the drive signal being emitted by an arbitrary waveform generator (8 GSa/s). The amplitude of these pulses during a roundtrip can additionally be controlled by another intensity modulator (IM2), which is also used to turn the pump signal on and off. The pump pulses first undergo second harmonic generation (SHG) inside a periodically poled lithium niobate (PPLN) waveguide. This frequency doubled pulsed train is then coupled into a second PPLN waveguide where it undergoes a halfharmonic generation process and is then injected into the cavity. This nonlinear process acts as a phase-sensitive amplifier (PSA). Above the threshold, the DOPOs will have a phase of either *φ*_*j*_ = 0 or *φ*_*j*_ = *π* relative to the pump pulses. Due to this binary phase, each DOPO can act as an independent spin. To implement the coupling term of the Ising Hamiltonian, pulses are coupled out of the cavity and their in-phase quadrature component $\tilde c_i$ is measured by using balanced homodyne detection (BHD). The amplitude is converted to a digital signal and processed inside an FPGA module, where the intensity of the feedback signal $\tilde f_i$ is calculated from the implemented coupling topology *J*_*ij*_ via:

2 $$\tilde f_i = - r\mathop {\sum}\limits_j {\kern 1pt} J_{ij}\tilde c_j.$$

During the measurement, we track the time evolution of the in-phase quadrature component by storing the FPGA data from the digitized BHD measurement. After each DOPO has propagated through the 1 km long cavity, a second train of injection pulses is modulated from the feedback signal by an intensity modulator (IM3) and injected into the cavity to interfere with the respective DOPO pulses. The push pull modulator is biased to *V*_*π*_ and changes the in-phase quadrature component. Negative feedback signals thus result in an optical phase of *π*, while positive feedback signals have an optical phase of 0. Arbitrary coupling topologies with up to *N*^2^ links can be implemented with this electro-optical measurement feedback scheme, which is a significant advantage over other analog computing schemes. In total, 5056 DOPOs are circulated inside the cavity, from which *N* = 2048 pulses can be used for calculation. We implement the 2D Ising model in a 44 by 44 grid with bidirectional nearest neighbor coupling and periodic boundary conditions with a coupling strength of *J*_*ij*_ = −1, resulting in a total of 1936 spins with 3872 bidirectional edges. The remaining 3008 pulses are uncoupled and used to phase-lock the cavity. For analysis of the correlation length, the spatial autocorrelation is estimated by shifting the solution along the grids two symmetry axes and fitting the autocorrelation with an exponential fit.

### Numerical simulation of DOPO network

The behavior of a DOPO network can be captured by *c*-number stochastic differential equations^17,18^, which model the in-phase amplitude *c*_*i*_ and quadrature amplitude *s*_*i*_ of the electric field for each DOPO:

3 $$\frac{{{\mathrm{d}}c_i}}{{{\mathrm{d}}t}} = \left( { - 1 + p - c_i^2 - s_i^2} \right)c + \frac{1}{{A_s}}\sqrt {c_i^2 + s_i^2 + \frac{1}{2}} \frac{{{\mathrm{d}}W_i}}{{{\mathrm{d}}t}}$$

4 $$\frac{{{\mathrm{d}}s_i}}{{{\mathrm{d}}t}} = \left( { - 1 - p - c_i^2 - s_i^2} \right)s + \frac{1}{{A_s}}\sqrt {c_i^2 + s_i^2 + \frac{1}{2}} \frac{{{\mathrm{d}}W_i}}{{{\mathrm{d}}t}}.$$

The pump strength of the master laser is described by the scale-less pump factor *p*, where *p*_*th*_ = 2 is the threshold value. When the optical pump is turned on, the DOPO amplitude will increase from the squeezed vacuum state, modeled by the Gaussian white noise process d*W*. The cavity losses and the DOPO generation process are summarized by the saturation amplitude *A*_*s*_ = 70. The in-coupling of pulses in to and the out-coupling of pulses from the fiber cavity is modeled by simple transfer functions for optical beamsplitter. For the simulations, the equations are numerically integrated, by using a fourth-order Runge–Kutta algorithm.

### Monte Carlo simulations of 2D Ising model

We simulate the 2D Ising model by Markov chain Monte Carlo simulations using the Metropolis algorithm. The Metropolis algorithm is a local update algorithm, where only a single spin is independently flipped in each update^26^. If the overall energy is decreased by an update, the spin flip is accepted. If the energy is increased by Δ*E*, there is a certain probability that the spin will nonetheless remain flipped, as given by

5 $$p = 1 - {\hskip 2pt}{\mathrm{e}}\, \, \hat{\vphantom{\mathrm {e}}} {\hskip 2pt}{\left( { - {\mathrm{\Delta }}E{\mathrm{/}}kT} \right)}.$$

We choose to update the spins with a typewriter ordering, so that all the spins are updated sequentially. While this typically improves performance over randomly selected update sites for the 2D model, it also mimics the sequential updating by the injection pulses in the coherent Ising machine.

## Data Availability

The authors declare that all relevant data are included in the manuscript. Additional data are available from the corresponding author upon reasonable request.
